# Feeble-Minded Children

**Published:** 1900-03-10

**Authors:** 


The Hospital

A Journal of

The Medical Sciences and Hospital Administration.

Vol. XXVII.—No. 702.]

Edited by Sir Henry Burdett, K.C.B., and Solomon C. Smith, M.D., M.R.C.P.

[March 10, 1900.

### FEEBLE-MINDED CHILDREN.

An interesting and instructive meeting was held on the 1st inst. at the house of the president, Earl Egerton of Tatton, to receive the annual report of the Council of the "Childhood Society," a society established, as its name implies, to promote the scientific study of the mental and physical conditions of children. It owes its origin mainly to the very extensive series of observations commenced and carried on by Dr. Francis Warner, the late Dr. Langdon Down, and the late Sir Douglas Galton, with regard to the innumerable intermediate steps which may separate a child of the highest intelligence from a child whose intelligence is not sufficient to enable it to profit by instruction; observations which may be said to show that, in many instances which it would hardly be possible to put together under any distinctive name, there exists a difficulty in the reception of teaching which renders the child unable to hold its own in an ordinary school, but which, by a proper adaptation of means to ends, may so far be overcome that, after a longer or shorter period, the subject becomes capable of learning with others in the ordinary way. The causes of such comparative incapacity may, as a rule, be referred to some manifest physiological or pathological condition. In some children there may be a greater or less defect of hearing, in some a greater or less defect of sight; and, in the unfortunate victims of inherited syphilis, these defects are frequently met with in combination, each of them intensifying the disability produced by the other. In some there is defect of memory. In some there may be simple slowness of reaction to nerve stimuli, so that a visual or an auditory impression requires a period of time in excess of the normal before it can excite a sensation or an idea; and the sensation or the idea, when excited, requires a period in excess of the normal before it goes out through the ordinary channels, as speech or as action. The child so constituted will always be behindhand; and the child who is always behindhand soon becomes too much discouraged to maintain a contest against its classmates in which it must always be handicapped by a natural disadvantage, and in which its slowness is too often attributed to obstinacy or to stupidity. The report of the Council of the society, which was taken as read at the meeting, sets forth that, in an examination of 100,000 school children, taken from all classes of the community, about 1·6 per cent. were observed to be either physically or mentally deficient to a marked extent, so as to profit little by ordinary education, and to require special separate classes in order to give them a reasonable chance of becoming useful citizens, and in order, also, to remove them from the ordinary schools, in which their presence is a hindrance to the other scholars. The Rev. T. W. Sharpe, C.B., the Chairman of Council of the society, and late H.M. Senior Inspector of Schools, in moving the adoption of the report, declared that the necessity of the case could only be met by a system of small classes for the instruction of defective children, classes which should on no account exceed 20 in number, and which would be much more effective if limited to 12; and which, in many instances, might with the greatest advantage be conducted in establishments which should at the same time be temporary "homes" for the pupils. He expressed a strong sense of the inutility, or worse, of large "institutions," on the ground that what was required was the personal influence on each child of a teacher whose heart was in the work, and whose affections were not diluted, and rendered practically inoperative, by the demands made upon them by numbers. Mr. Sharpe went on to say that the experiments hitherto made in this direction had been exceedingly satisfactory in their results, and that large numbers of children who, if left to ordinary schools and ordinary methods, would never have been able to become useful members of society, had, after a short period of special instruction, so far improved as to be able to take their places in a class with others, and had ultimately been able to learn some trade or occupation by which, even if not wholly able to support themselves, they were nevertheless withdrawn from being absolute burdens upon their relatives or upon charity, and were enabled to live comparatively happy and useful lives. That this should be so is in complete accordance with all that physiology teaches with regard to the development and activity of the nervous system, and while our knowledge of that system seems likely to increase year by year, and our power of guiding or controlling its operations to increase in similar proportion, it is eminently satisfactory to find the highest "practical" educational authorities coming to recognise that the "preparations made during our youth for the sequel of our lives" can only be made effectually when they are founded upon due consideration of the needs and peculiarities of the organism through which the phenomena of life are displayed. It should be noted that an Act passed during the last session of Parliament gives power to County Councils and other authorities to contribute towards the special education of feeble-minded children from public funds, and it is hoped that in the near future this power, under adequate medical supervision, will be liberally and extensively exercised.

